# Haemodynamic, hormonal and renal actions of osteocrin in normal sheep

**DOI:** 10.1113/EP091826

**Published:** 2024-06-18

**Authors:** Nicola J. A. Scott, Timothy C. R. Prickett, Christopher J. Charles, Eric A. Espiner, A. Mark Richards, Miriam T. Rademaker

**Affiliations:** ^1^ Department of Medicine, Christchurch Heart Institute University of Otago Christchurch Christchurch New Zealand; ^2^ Cardiovascular Research Institute, National University Health Systems Centre for Translational Medicine Singapore Singapore

**Keywords:** cAMP, cGMP, natriuretic peptides, osteocrin metabolism, osteocrin molecular forms

## Abstract

Osteocrin (OSTN) is an endogenous protein sharing structural similarities with the natriuretic peptides [NPs; atrial (ANP), B‐type (BNP) and C‐type (CNP) NP], which are hormones known for their crucial role in maintaining pressure/volume homeostasis. Osteocrin competes with the NPs for binding to the receptor involved in their clearance (NPR‐C). In the present study, having identified, for the first time, the major circulating form of OSTN in human and ovine plasma, we examined the integrated haemodynamic, endocrine and renal effects of vehicle‐controlled incremental infusions of ovine proOSTN (83–133) and its metabolism in eight conscious normal sheep. Incremental i.v. doses of OSTN produced stepwise increases in circulating concentrations of the peptide, and its metabolic clearance rate was inversely proportional to the dose. Osteocrin increased plasma levels of ANP, BNP and CNP in a dose‐dependent manner, together with concentrations of their intracellular second messenger, cGMP. Increases in plasma cGMP were associated with progressive reductions in arterial pressure and central venous pressure. Plasma cAMP, renin and aldosterone were unchanged. Despite significant increases in urinary cGMP levels, OSTN administration was not associated with natriuresis or diuresis in normal sheep. These results support OSTN as an endogenous ligand for NPR‐C in regulating plasma concentrations of NPs and associated cGMP‐mediated bioactivity. Collectively, our findings support a role for OSTN in maintaining cardiovascular homeostasis.

## Abstract (highlights)

- **What is the central question of this study?**
  What role does osteocrin (OSTN) have in integrated cardiovascular, renal and neurohumoral function in normal health?
- **What is the main finding and its importance?**
  Osteocrin is structurally similar to natriuretic peptides (NPs) that play a crucial role in cardiovascular homeostasis and binds to the NP receptor‐C (NPR‐C). Stepped doses of OSTN raised plasma atrial NP, B‐type NP, C‐type NP and cGMP and reduced arterial and venous pressures. Collectively, these findings provide strong evidence that increased NP levels result from competitive displacement from NPR‐C, not from increased NP secretion, suggesting that OSTN might play a role in maintaining cardiovascular homeostasis.

## 1 INTRODUCTION

The natriuretic peptides (NPs) are potent and important regulators of the cardiovascular system. This family of structurally and functionally related peptides includes atrial (ANP), B‐type (BNP) and C‐type (CNP) NPs. Both ANP and BNP are best known for their role in maintaining pressure/volume homeostasis by promoting renal sodium and water excretion and stimulating vasodilatation, along with the ability to inhibit the renin–angiotensin–aldosterone and sympathetic nervous systems, while the paracrine action of CNP complements the endocrine action of ANP and BNP (Kuhn, 2016; Levin *et al.*, 1998; Potter, 2011; Sarzani *et al.*, 2022). The NPs exert their actions primarily through interaction with their cell surface receptors, NP‐receptor‐A (NPR‐A) and NPR‐B [both coupled to the guanylyl cyclase (GC)– cGMP signal transduction pathway]. A third receptor, NPR‐C, lacks the intracellular GC domain and acts as a ‘clearance’ receptor, controlling local concentrations of all the NPs through internalization and degradation (Potter, 2011).

Osteocrin (OSTN, synonymous with musclin) is a novel secreted peptide that has recently been proposed as a new member of the NP family. This peptide, originally identified in fetal bone (Thomas *et al.*, 2003) and skeletal muscle (Nishizawa *et al.*, 2004), is processed intracellularly from the prohormone (Nishizawa et al., 2004) to smaller bioactive forms of 5 or 10 kDa. Although OSTN has been measured in adult human plasma in the low picomolar range (Chen *et al.*, 2017), the circulating form has yet to be characterized fully (Kita *et al.*, 2009). To date, no specific receptor for OSTN has been identified, and functions of the peptide and its regulation in humans remain to be clarified. However, OSTN contains two amino acid (aa) sequence motifs showing close homology to the NPs, specifically in the region within the 17 aa ring structure essential for receptor binding (Papaleo *et al.*, 2010). These similarities in sequence result in OSTN binding competitively to the NP clearance receptor NPR‐C (but not to receptors NPR‐A and NPR‐B; Kita et al., 2009), thereby reducing NP clearance and prolonging NP bioactivity (Thomas et al., 2003). Of note, NPR‐C is by far the most abundant of the NP receptors (accounting for >95% of the total population) and is located in high density in the heart, lung, adipose tissue and systemic vasculature, which are sites relevant to observed NP activity (Potter *et al.*, 2006).

Studies in mice have subsequently confirmed the competitive interaction of OSTN with ANP in binding to NPR‐C and showed that when administered at high dosage, OSTN increases plasma levels of ANP in association with reductions in blood pressure (Kita et al., 2009; Miyazaki *et al.*, 2018). Notably, in a murine model of cardiac overload, cardiac overexpression of OSTN results in an improvement of cardiac performance, which the authors attributed to an increase in CNP (Szaroszyk *et al.*, 2022). Collectively, these studies suggest that OSTN might be an endogenous ligand for NPR‐C capable of regulating plasma concentrations of the NPs, in addition to local bioavailability and activity in tissues targeted by the NPs. Although the physiological effects of OSTN have been ascribed largely to reduced NPR‐C‐dependent NP clearance, there is some evidence of direct biological activity mediated by this receptor (via G_i_‐inhibitory/regulatory proteins and several intracellular signalling pathways, including adenylyl cyclase/cAMP signal transduction; Anand‐Srivastava, 2005; Nishida *et al.*, 2021), including cardiovascular effects (Moyes *et al.*, 2020) and renal tubular sodium reabsorption (Shao *et al.*, 2021).

To date, there are no studies that have examined these putative molecular interactions of OSTN with physiological concentrations of all three NPs nor the integrated effects of these interactions in conscious animals. In the present study, after showing that the major circulating form of OSTN is the 5 kDa peptide in both sheep and humans, we have examined the impact of stepped ascending doses of this peptide on circulating NP (ANP/BNP/CNP) and cGMP levels, together with haemodynamic, neurohumoral and renal responses, in healthy conscious sheep.

## 2 MATERIALS AND METHODS

### 2.1 Ethical approval

All animal procedures were carried out in compliance with the ARRIVE (Animal Research: Reporting of In Vivo Experiments) guidelines and approved by the Animal Ethics Committee of the University of Otago, Christchurch (#AUP 20‐121) [in accordance with the Animal Welfare Act 1999, including the Animal Welfare Amendment Act No. 2 (2015)]. The authors understand the ethical principles under which the journal operates and confirm that their work complies with these principles.

### 2.2 Surgical preparation

Eight Coopworth ewes (4–6 years; 42–60 kg; Lincoln University Farm, New Zealand) were instrumented under general anaesthesia (induced by 0.5 mg/kg diazepam i.v. and 4 mg/kg ketamine i.v. and maintained with a mixture of isoflurane and oxygen) and using approved peri‐ and postoperative antibiotics (20 mg/kg cephazolin i.v.; 6.6–20 mg/kg oxytetracycline i.v.) and analgesia (4 mg/kg carprofen i.v.). Sheep were instrumented via a 50‐mm‐long neck incision, allowing cannulation of the left carotid artery (16 gauge Angiocath; Becton Dickinson, Sandy, UT, USA) for direct measurement of mean arterial pressure (MAP) and heart rate (HR). A polyethylene catheter was placed in the left jugular vein for blood sampling and recording of central venous pressure (CVP). A Swan–Ganz thermodilution catheter (Edwards Life Sciences, Irvine, CA, USA) was placed in the pulmonary artery via the jugular vein for the measurement of cardiac output (CO). The urinary bladder was catheterized per urethra for timed urine collections (Charles, Espiner, Nicholls *et al.*, 1996). Animals recovered for ≥5 days before commencing the study protocol.

During experiments, all animals were housed in metabolic cages in an air‐conditioned, light‐controlled room and received a diet of lucerne chaff and food pellets providing ∼75 mmol sodium and ∼150 mmol potassium per day, with free access to water.

### 2.3 Study protocol

Sheep received i.v. infusions of increasing doses of ovine proOSTN (83–133), the putative 51 aa circulating peptide (at 2.5, 25 and 250 pmol/kg/min for 1 h each; Mimotopes, Melbourne, Victoria, Australia) and a vehicle control (0.9% saline) administered on separate days, ≥1 day apart, in a balanced random order.

Haemodynamic measurements [HR, MAP, CVP and CO, and calculated total peripheral resistance (CTPR = MAP/CO)] were recorded at 30 min intervals in the hour preceding the first dose (baseline) and at 15, 30, 45 and 60 min during each infusion. Additional measurements were made over the 120 min period following cessation of the highest‐dose infusion in order to measure peptide half‐life. Measurements were determined by online computer‐assisted analysis (PowerLab Systems; ADInstruments, Dunedin, New Zealand) and made with the animals standing quietly in their metabolic crates (Charles, Espiner, Nicholls *et al.*, 1996).

Venous blood samples were obtained at 30 min and immediately preceding commencement of the first infusion (baseline) and at 30  min intervals for the duration of the study day. Samples were taken into tubes on ice, centrifuged at 4°C and stored at −20°C before assaying. Following extraction over C18 SepPac cartridges (Waters Corp., Milford, MA, USA), plasma concentrations of cGMP (Charles *et al.*, 1990), ANP (Charles et al., 1990), BNP (Pemberton *et al.*, 1997), CNP (Charles *et al.*, 1995) and N‐terminal proCNP (NTproCNP) (Wilson *et al.*, 2015), were measured by radioimmunoassay (RIA) as previously described. Non‐extracted plasma renin activity (PRA) (Dunn & Espiner, 1976) and aldosterone (Lun *et al.*, 1983) were measured as previously described. Cyclic AMP was measured by enzyme‐linked immunosorbent assay (catalogue no. ADI‐900‐163; Enzo Life Sciences, Farmingdale, NY, USA). For each analyte, all samples from individual animals were measured in the same assay to avoid interassay variability. Haematocrit concentrations were measured with every blood sample taken.

Blood samples for analysis of plasma sodium, potassium and creatinine concentrations were taken immediately before the first dose (baseline), then hourly throughout the study. Urine volume and samples for the measurement of urine cGMP, urine cAMP, sodium, potassium and creatinine excretion were collected hourly throughout the study protocol. To estimate the contribution of renal tissues to urine cGMP/cAMP excretion, nephrogenous cGMP/cAMP was calculated using the formula [urine cG(A)MP − (glomerular filtration rate × plasma cG(A)MP/urine volume × min] (Hirata *et al.*, 1990).

Osteocrin concentrations in the infusate were measured to calculate the infusion rate and metabolic clearance rate (MCR) of the peptide (MCR = infusion rate/plateau − baseline level). On the OSTN treatment day, at the completion of the 250 pmol/kg/min infusion, additional plasma samples were taken at 1, 2, 3, 5, 7.5, 10 and 15  min for determination of OSTN plasma half‐life.

### 2.4 Euthanasia

At the completion of the study, sheep were killed with an i.v. overdose (150 mg/kg) of pentobarbitone sodium (300 mg/mL; Provet NZ, Auckland, New Zealand). Death was confirmed by auscultation.

### 2.5 Development of radioimmunoassay for OSTN

#### 2.5.1 Peptides

**FIGURE 1:**
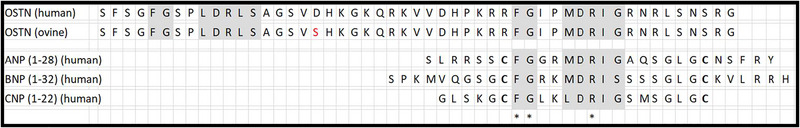
Alignment of human and ovine osteocrin (OSTN) amino acid sequences from residue 83 to 133 and homology to the natriuretic peptides (NPs). Human and ovine OSTN sequences differ at residue 97, indicated in red. The blocks of grey shading highlight the two homologous regions of OSTN for binding to the NP receptor‐C (NPR‐C). An asterisk (*) denotes the residues important for NP binding to NPR‐C conserved across all family members (Kita et al., 2009; Moffatt et al., 2007).

Human proOSTN (83–133) and human proOSTN (83–112) were obtained from Phoenix Pharmaceuticals (Burlingame, CA, USA; catalogue nos 028‐68 and 028‐61, respectively). Ovine proOSTN (83–133) was synthesized by Chiron Technologies, Australia. Given that human and ovine proOSTN (83–112) sequences differ only at aa 100 (Figure 1), we expected the antiserum to cross‐react with proOSTN (83–112) from both species.

#### 2.5.2 Plasma samples

Samples for the purposes of initial testing and analytical validation of the OSTN assay were obtained from Christchurch Heart Institute laboratory staff (*n* = 5, aged 23–58 years) in accordance with the laboratory policy and an institutional consent form for blood and urine donation (Section 12.2.13, Issue 7, September, 2020). Volunteers could withdraw at any time, and samples obtained were used for no purpose other than OSTN testing. Collected plasma was rapidly centrifuged and stored frozen prior to assay. To assess fluctuations throughout the day and the impact of food intake, repeated hourly sampling for measurement of OSTN was undertaken over a 5 h period. Ovine plasma samples were obtained from a laboratory biorepository of normal healthy sheep, where residual plasma was available and had been stored at −80°C. EDTA plasma samples (1 mL) were extracted using Sep‐Pak C18 cartridges (Waters Corp., Milford, MA, USA). Bound peptides were eluted from the cartridges with 2 mL of 80% isopropanol in 0.1% trifluoroacetic acid into collection tubes containing 10 μL of 0.1% Triton X‐100. The eluant was collected, dried under an air stream at 37°C, and frozen at −20°C. Dried extracts were resuspended in 0.5 mL assay buffer prior to RIA.

#### 2.5.3 Osteocrin radioimmunoassay

Human proOSTN (83–112) (5 mg) was iodinated using 0.5 mCi Na^125^I in the presence of 10 mg chloramine‐T in 5 mL 0.5 pmol/L phosphate buffer, pH 7.5, for 20 s, followed by the addition of 50 mg cysteine, 25 mg BSA and 20 mg KI in 100 mL buffer. The resulting mixture was loaded onto a 10 cm RP300 Brownlee column and eluted with a gradient from 0% to 60% acetonitrile in 49 mmol/L KH_2_PO_4_ buffer, pH 2.9, over 30 min at 1 mL/min, collecting 0.5 mL fractions. The fraction containing the major peak of radioactive [^125^I]‐proOSTN (83–112) was used in the RIA. Peptide standards were made from synthetic human proOSTN (83–133) taking into account the purity data supplied. All standards, sample extracts, antisera and tracer solutions were made up in pH 7.4 assay buffer (0.1% BSA in 0.1 M phosphate buffer). Fifty microlitres of sample extract or 1−1000 pmol/L of human proOSTN (83–133) standard (all in duplicate) were incubated with 50 μL antiserum H‐028‐68 (Phoenix Pharmaceuticals) at a dilution of 1:20,000 for 24 h at 4°C, followed by addition of 50 μL of tracer solution [^125^I]‐proOSTN (83–112) containing 3000 cpm for an additional 24 h at 4°C. Bound and free [^125^I]‐proOSTN (83–112) were separated by a solid‐phase second antibody method (Sac‐Cell, Donkey‐Anti Rabbit; IDS, UK), and the radioactivity in the bound [^125^I]‐proOSTN (83–112) pellet was measured in a PerkinElmer Wizard2 10‐Detector Gamma Counter. Ovine concentrations of OSTN were extrapolated from the human standards, taking into account ovine proOSTN (83–133) cross‐reactivity. Ovine proOSTN (83–112) cross‐reactivity in the ANP, BNP and CNP RIA was <0.005%.

### 2.6 Statistical analysis

**FIGURE 2:**
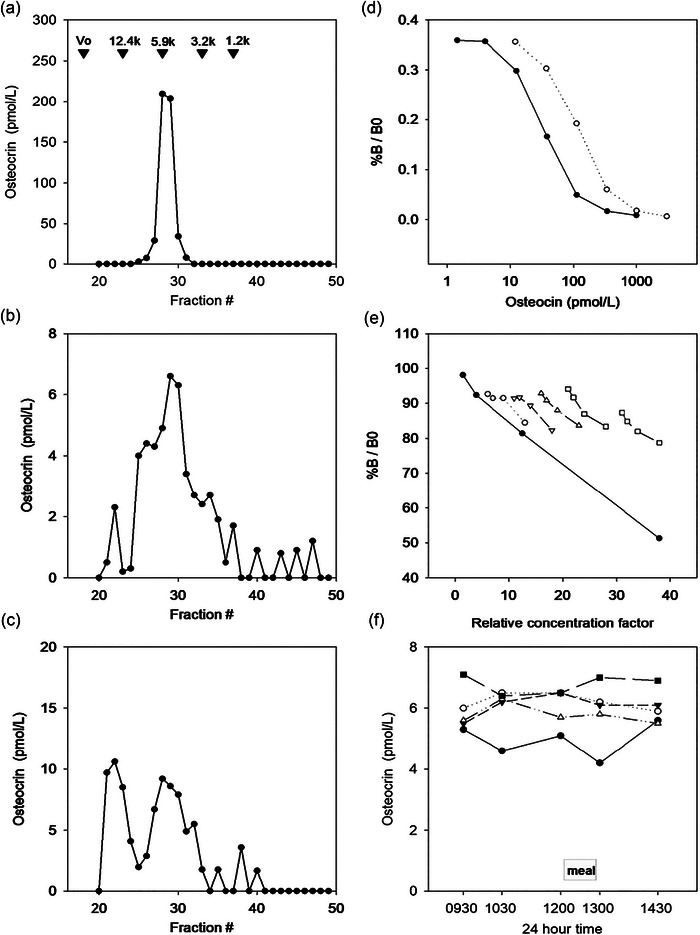
Osteocrin (OSTN) radioimmunoassay. (a–c) Size exclusion HPLC–radioimmunoassay (RIA) profile of the following: (a) synthetic human proOSTN (83–133) (column void volume and elution position of molecular weight markers are shown by arrows); (b) ovine plasma extract; and (c) human plasma extract. (d) Serial dilutions of human proOSTN (83–133) standard (filled circles) and ovine proOSTN (83–133) standard (open circles). (e) Serial dilutions of human proOSTN (83–133) standard (filled circles) and five different healthy adult plasma extracts (open symbols). (f) The variation in circulating concentrations of OSTN during the day and after consumption of a lunchtime meal for five healthy adults.

**FIGURE 3:**
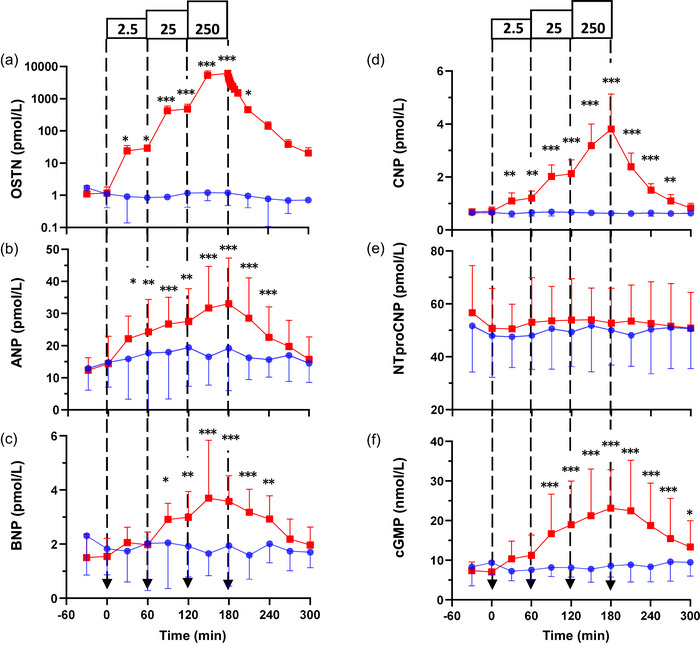
Hormone responses to incremental infusions of osteocrin (OSTN; red squares) at 2.5, 25 and 250 pmol/kg/min and a vehicle control (blue circles) in normal conscious sheep (*n* = 8). Infusion of OSTN significantly increased plasma levels of the following: (a) OSTN (expressed on a log_10_ scale; *P <* 0.001); (b) atrial natriuretic peptide (ANP; *P <* 0.001); (c) B‐type natriuretic peptide (BNP; *P <* 0.001); (d) C‐type natriuretic peptide (CNP; *P <* 0.001); and (f) cGMP (*P <* 0.001). (e) Infusion of OSTN had no significant effect on plasma N‐terminal proCNP (NTproCNP; *P =* 0.064). Results are expressed as the mean ± SD. Individual time points significantly different from time‐matched vehicle control (Fisher's protected LSD from two‐way ANOVA) are indicated by *P < 0.05, **P < 0.01 and ***P < 0.001.

**FIGURE 4:**
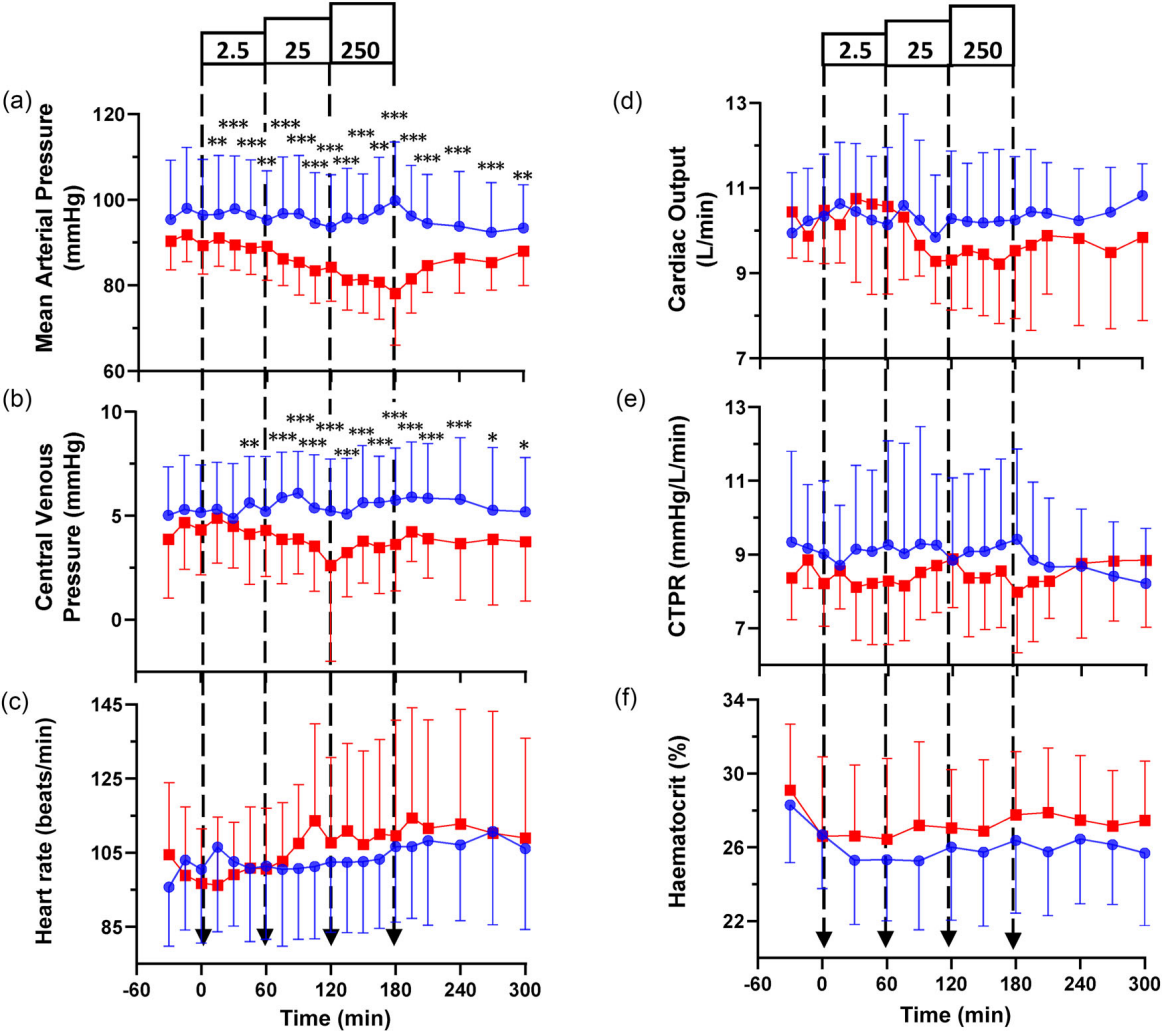
Haemodynamic and haematocrit responses to incremental infusions of osteocrin (OSTN; red squares) at 2.5, 25 and 250 pmol/kg/min and a vehicle control (blue circles) in normal conscious sheep (*n* = 8). (a, b) Infusion of OSTN significantly reduced mean arterial pressure (*P <* 0.001; a) and central venous pressure (*P = *0.003; b). (c–f) Infusion of OSTN had no significant effect on heart rate (*P = *0.140; c), cardiac output (*P* = 0.546; d), calculated total peripheral resistance (CTPR; *P =* 0.356; e) or haematocrit (*P* = 0.360; f). All haemodynamic responses are expressed as the mean ± SD. Individual time points significantly different from time‐matched vehicle control (Fisher's protected LSD from two‐way ANOVA) are indicated by *P < 0.05, **P < 0.01 and ***P < 0.001.

Results are expressed as the mean ± SD. To test for the effects of OSTN, control and OSTN study limbs were compared with repeated‐measures ANOVA (SPSS v.28; SPSS, Chicago, IL, USA), with time and treatment as within‐subject factors. Where significant differences were identified by ANOVA, the level of significance at individual time points was determined by Fisher's protected least‐significant difference (LSD) tests (Figures 2, 3, 4; Table 1). Time × treatment interactions are quoted in the text, table and figures (unless stated otherwise). Significance was assumed at *P <* 0.05.

**TABLE 1:** Effects of osteocrin administration in normal sheep.

Parameter	Group	Baseline	OSTN	OSTN	OSTN			
			2.5 pmol/kg/min	25 pmol/kg/min	250 pmol/kg/min			
		0 h	1 h	2 h	3 h	4 h	5 h	*P*‐value
Plasma cAMP	Control	10.4 ± 4.9	9.5 ± 3.9	9.2 ± 4.0	9.9 ± 4.0	9.0 ± 4.7	12.2 ± 4.8	
(pmol/L)	OSTN	13.5 ± 3.7	11.5 ± 4.8	11.6 ± 3.0	12.9 ± 6.5	10.4 ± 7.1	10.7 ± 6.1	0.278
Plasma aldosterone	Control	206 ± 144	282 ± 439	188 ± 192	162 ± 106	192 ± 184	207 ± 197	
(pmol/L)	OSTN	208 ± 148	199 ± 156	176 ± 137	167 ± 157	153 ± 142	192 ± 130	0.338
Plasma renin activity	Control	0.112 ± 0.040	0.105 ± 0.055	0.106 ± 0.075	0.099 ± 0.060	0.141 ± 0.073	0.205 ± 0.122	
(nmol/L/h)	OSTN	0.168 ± 0.071	0.153 ± 0.070	0.153 ± 0.065	0.155 ± 0.075	0.186 ± 0.109	0.241 ± 0.181	0.208
Plasma sodium	Control	145.9 ± 1.6	147.5 ± 2.6	147.1 ± 2.2	147.0 ± 1.7	147.5 ± 1.7	148.3 ± 2.0	
(mmol/L)	OSTN	145.8 ± 1.6	147.0 ± 2.6	147.0 ± 2.5	146.9 ± 2.4	147.1 ± 2.2	146.5 ± 2.1	0.226
Plasma potassium	Control	4.2 ± 0.3	4.0 ± 0.4	4.1 ± 0.4	4.3 ± 0.4	4.4 ± 0.4	4.3 ± 0.2	
(mmol/L)	OSTN	4.0 ± 0.3	3.8 ± 0.3	4.0 ± 0.5	4.0 ± 0.5	4.1 ± 0.6	4.3 ± 0.6	0.620
Plasma creatinine	Control	76.6 ± 12.4	75.1 ± 10.4	74.6 ± 11.3	75.3 ± 10.6	75.4 ± 11.1	75.0 ± 10.9	
(μmol/L)	OSTN	79.3 ± 12.2	76.6 ± 7.5	75.6 ± 6.9	76.4 ± 8.5	75.6 ± 8.9	74.6 ± 8.5	0.740
Urine potassium	Control	15.2 ± 4.5	14.2 ± 3.9	14.5 ± 4.2	9.7 ± 5.4	9.4 ± 5.1	10.6 ± 4.0	
(mmol/h)	OSTN	16.3 ± 4.5	15.6 ± 3.5	12.7 ± 4.9	9.9 ± 3.6	7.9 ± 3.4	7.4 ± 3.6	0.274
Urine creatinine	Control	0.513 ± 0.100	0.528 ± 0.100	0.585 ± 0.100	0.508 ± 0.100	0.506 ± 0.100	0.517 ± 0.200	
(mmol/h)	OSTN	0.544 ± 0.200	0.635 ± 0.100	0.593 ± 0.100	0.586 ± 0.200	0.533 ± 0.200	0.542 ± 0.100	0.749
Creatinine clearance	Control	0.115 ± 0.032	0.120 ± 0.030	0.132 ± 0.025	0.115 ± 0.031	0.114 ± 0.026	0.119 ± 0.054	
(L/min)	OSTN	0.120 ± 0.059	0.139 ± 0.030	0.132 ± 0.035	0.129 ± 0.043	0.118 ± 0.036	0.115 ± 0.028	0.807
Urine cAMP	Control	763 ± 404	673 ± 334	754 ± 257	608 ± 542	571 ± 469	742 ± 797	
(pmol/h)	OSTN	840 ± 524	674 ± 367	699 ± 322	549 ± 211	452 ± 219	891 ± 1136	0.984
Nephrogenous cAMP	Control	8184 ± 5409	6486 ± 5416	4498 ± 3251	3611 ± 3576	3526 ± 2139	3817 ± 1978	
(pmol/L)	OSTN	9423 ± 7087	5121 ± 4175	3204 ± 1657	2771 ± 1972	3007 ± 1891	10,142 ± 16,574	0.947
Nephrogenous cGMP	Control	2276 ± 4033	1634 ± 2773	1553 ± 3062	1311 ± 2211	1090 ± 2492	1702 ± 2638	
(pmol/L)	OSTN	2086 ± 3795	2796 ± 5690	1734 ± 2960	3320 ± 6541	2981 ± 4758	2362 ± 4272	0.139

*Note*: Values are the mean ± SD. Responses following hourly stepwise infusion of vehicle control or osteocrin (OSTN) in eight normal sheep. Baseline values represent the mean of samples taken over the period prior to treatment.

## 3 RESULTS

The developed OSTN RIA had an ED_50_ value of 30 pmol/L and a detection limit (2 SD from zero) of 2 pmol/L. The size exclusion HPLC–RIA profile of synthetic human proOSTN (83–133) (Figure 2a) showed a peak elution at fractions 28/29 (5 kDa). The predominant peak of immunoreactivity observed in the ovine (Figure 2b) and human (Figure 2c) SE‐HPLC profiles is likely to represent the 5 kDa form identified by Moffatt et al. (2007). Human and ovine OSTN standards diluted out in parallel (Figure 2d), as did samples from five healthy adults (Figure 2e). The ovine sequence differs by one aa in the region corresponding to that used for the RIA tracer human proOSTN (83–112), and the cross‐reactivity of the ovine standard in the RIA was 31%. Recovery of spiked OSTN [human proOSTN (83–133) at 20 pmol/L] from human plasma samples ranged from 71% to 76% (*n* = 5). Intra‐ and interassay coefficients of variation were 4.2% and 9.3%, respectively, at 6.5 pmol/L. No significant variation in circulating concentrations of OSTN was observed following repeated measurements in human volunteers throughout the day or throughout a meal (Figure 2f).

All sheep experiments were completed without mishap, and data collection was complete (*n* = 8 sheep). Incremental infusions of OSTN increased plasma concentrations of the peptide in a dose‐dependent manner (*P* < 0.001; Figure 3a). Compared with control data where plasma OSTN levels remained stable (at ∼1 pmol/L), plasma OSTN levels rose acutely in a stepwise fashion with the stepped infusion protocol, reaching 28.7 ± 8.0, 479.5 ± 194.6 and 6094.6 ± 750 pmol/L at the end of 2.5, 25 and 250 pmol/kg/min 60 min infusions, respectively. On termination of the highest‐dose OSTN infusion (250 pmol/kg/min), plasma OSTN levels fell promptly, although did not fully return to pre‐infusion levels over the following 120 min. Osteocrin plasma half‐life in normal sheep was 5.7 ± 1.8 min.

In response to increases in plasma OSTN, circulating levels of ANP, BNP and CNP also increased in a stepwise fashion compared with the vehicle control (all *P* < 0.001; Figure 3b–d). Plasma ANP levels were constant over the vehicle control day at ∼16.5 ± 10.0 pmol/L, and in response to the stepped OSTN infusions they rose from a baseline of 14.4 ± 8.5 pmol/L to 24.3 ± 10.1, 27.6 ± 10.2 and 33.0 ± 14.2 pmol/L at the end of each 60 min incremental infusion (Figure 3b). Likewise, circulating BNP levels were stable throughout the vehicle control day at ∼1.9 ± 1.1 pmol/L, and rose from a baseline of 1.5 ± 0.7 pmol/L to 2.0 ± 0.5, 3.1 ± 0.9 and 3.9 ± 2.3 pmol/L at the end of each 60 min OSTN infusion (Figure 3c). Plasma CNP levels were constant over the vehicle control day at ∼0.64 ± 0.11 pmol/L, and rose from a baseline of 0.71 ± 0.20 pmol/L to 1.21 ± 0.30, 2.13 ± 0.50 and 3.81 ± 1.30 pmol/L in response to the stepped OSTN infusions (Figure 3d). There was no corresponding rise in levels of bioinactive NTproCNP (Figure 3e).

Plasma cGMP rose significantly and in a dose‐dependent manner in response to incremental OSTN infusions (*P *< 0.001; Figure 3f). The cGMP levels were stable over the vehicle control day at ∼8.42 ± 3.38 nmol/L, whereas concentrations rose from a baseline of 7.4 ± 2.2 nmol/L to 11.2 ± 5.1, 19.0 ± 11.0 and 23.1 ± 9.7 nmol/L at the end of each 60 min OSTN infusion (Figure 3f).

Infusion of OSTN did not result in significant changes in plasma cAMP, PRA, aldosterone or electrolyte levels compared with vehicle control (Table 1).

Haemodynamic responses to OSTN infusion are shown in Figure 4. Compared with time‐matched control data, MAP decreased in a dose‐dependent fashion in response to increasing doses of OSTN (*P* < 0.001; Figure 4a), falling progressively by ∼2, ∼7 and ∼12 mmHg, respectively, at the end of each infusion period compared with time‐matched vehicle control. The MAP returned gradually towards baseline levels over the subsequent 120 min following cessation of OSTN infusion. Administration of OSTN also reduced CVP (*P *= 0.003; Figure 4b), with significant reductions occurring in response to both the 25 and 250 pmol/kg/min infusions. Infusion of OSTN did not significantly alter HR, CO, CTPR or haematocrit in comparison to vehicle control (Figure 4c–f).

**FIGURE 5:**
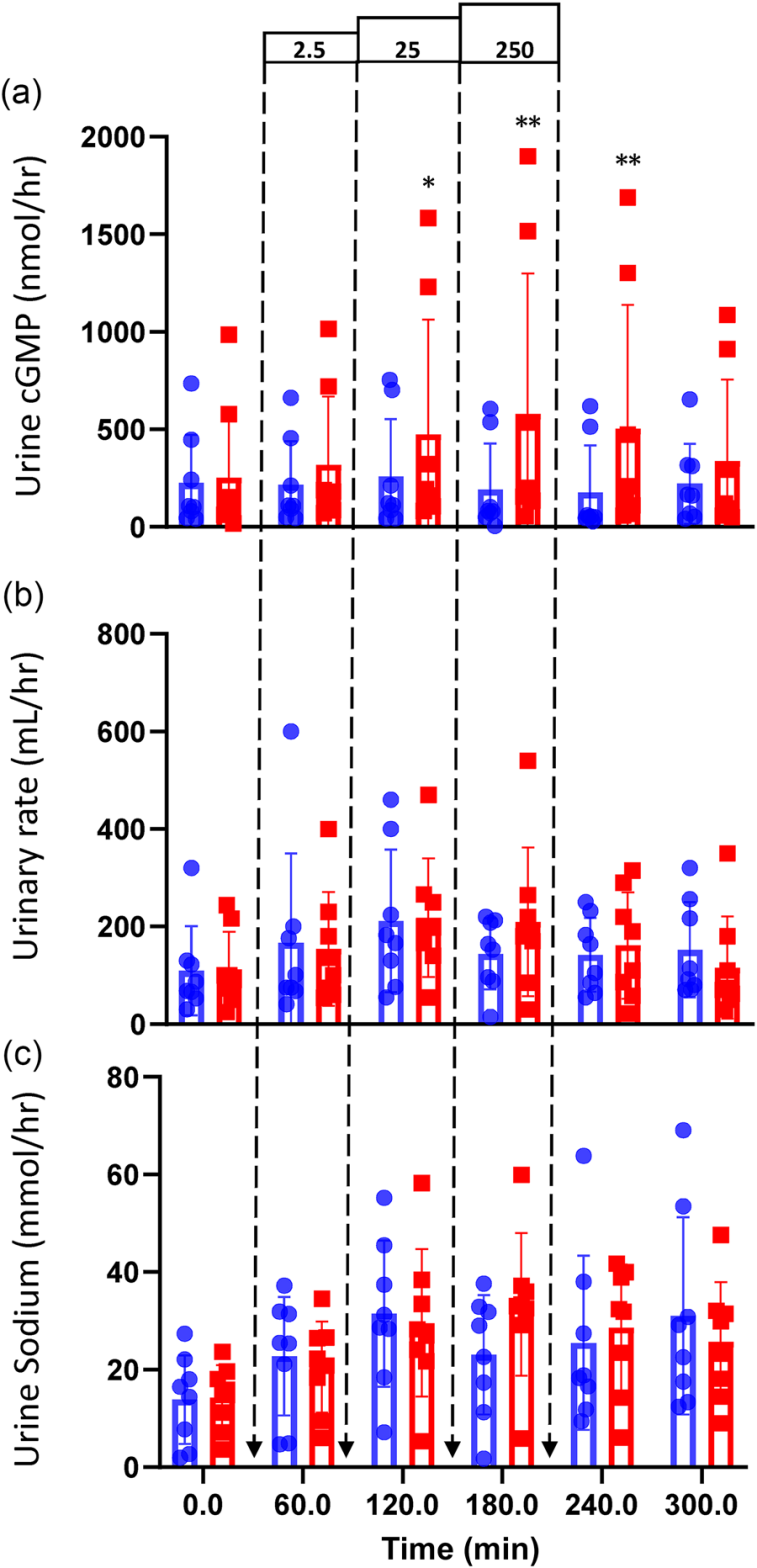
Renal responses to incremental infusions of osteocrin (OSTN; red bars and squares) at 2.5, 25 and 250 pmol/kg/min and a vehicle control (blue bars and circles) in normal conscious sheep (*n* = 8). (a) Infusion of OSTN significantly increased urine cGMP (*P *= 0.002). (b, c) Infusion of OSTN did not increase urinary rate (*P *= 0.467; b) or urine sodium excretion (*P* = 0.226; c). Renal responses are expressed as the mean ± SD and individual results plotted by symbol. Individual time points significantly different from time‐matched vehicle control (Fisher's protected LSD from two‐way ANOVA) are indicated by *P < 0.01 and **P < 0.001.

Osteocrin infusion increased urinary cGMP excretion in a dose‐dependent manner (*P* = 0.002; Figure 5a); however, at the doses administered, in healthy animals, it was neither diuretic nor natriuretic (Figure 5b, c). Urinary cAMP, potassium and creatinine excretion rates and creatinine clearance were similar on OSTN‐infusion and control days (Table 1). Neither nephrogenous cGMP nor cAMP was significantly different from control values during or after OSTN infusions (Table 1).

## 4 DISCUSSION

Although OSTN is a secreted protein (Kita et al., 2009; Thomas et al., 2003) and is reported to circulate in plasma at low picomolar concentrations (Chen et al., 2017; Subbotina *et al.*, 2015), the molecular forms of the peptide in plasma have not been characterized previously. Here, we provide evidence that the 5 kDa peptide [proOSTN (83–133)] is the predominant circulating form in both healthy humans and sheep. Using a sensitive RIA for this peptide in sheep, we have quantified the impact of dose‐dependent changes in OSTN on the dynamic changes in bioactive NP levels and the associated changes in cardiovascular and renal function in normal sheep. Increasing basal plasma OSTN from 1 pmol/L to stepped levels ranging from 28 to 6100 pmol/L evoked stepped and sustained increases in ANP, BNP and CNP, but not in the bioinactive product of proCNP secretion, NTproCNP. Time‐matched increases in plasma and urine cGMP during OSTN infusions, and the significant vasodepression (reduced MAP and CVP) without natriuresis, are consistent with the cardiorenal responses to exogenous NP infusions previously reported in normal sheep (Charles, Espiner, Richards *et al.*, 1996). Collectively, these findings provide strong evidence that the observed increase in NP levels results from competitive displacement from NPR‐C, not from increased NP secretion. They suggest that OSTN might play a role in maintaining cardiovascular homeostasis. Furthermore, absence of change in circulating (and urinary) cAMP suggests that the putative role of NPR‐C in stimulating G_i_‐binding proteins (Maack *et al.*, 1987; Moyes & Hobbs, 2019) plays a much lesser (if any) role in determining function compared with GC receptors, although any local effects at the tissue/cellular level cannot be determined from this study.

Actions of OSTN on muscle cells (Subbotina et al., 2015) and cartilage/bone (Moffatt et al., 2007; Thomas et al., 2003) have been studied, whereas few reports have examined the impact of the peptide on plasma concentrations of NPs and on cardiovascular and renal tissues. A crucial issue is the ability of OSTN, in tissues and in plasma, to impact NP concentrations in health or disease. In mice, constant infusion of the 10 kDa peptide [proOSTN (30–130)] at doses of 10 μg/kg/min (∼1000 pmol/kg/min) increased endogenous plasma ANP (by ∼15 pmol/L), similar to increments evoked by administration of the NPR‐C agonist C‐ANP‐(4–23) (Kita et al., 2009). In later studies by the same group (Miyazaki et al., 2018), infusions of the synthetic 10 kDa OSTN peptide increased plasma ANP and reduced blood pressure in a dose‐dependent manner. Osteocrin infused at rates of 1 μg/kg/min (100 pmol/kg/min) induced an increase in ANP to ∼15 pmol/L, compared with control (5 pmol/L), and was associated with a 10 mmHg decline in blood pressure. Plasma concentrations of OSTN were not reported. In the present study, the lowest infusion rate (2.5 pmol/kg/min) raised plasma OSTN from 1.0 to ∼30 pmol/L and increased circulating ANP (from 14 to 24 pmol/L), BNP (from 1.5 to 2.1 pmol/L) and CNP (from 0.7 to 1.2 pmol/L), resulting in the significant increase in plasma cGMP and reduction in arterial pressure within the first hour. Virtually identical increments of plasma ANP and CNP were reported in a mouse model overexpressing OSTN, but at vastly higher plasma concentrations of OSTN (∼10,000 pmol/L) (Miyazaki et al., 2018). Although the impact of the interaction between equimolar plasma concentrations of OSTN and NPs needs to be assessed, preferably at levels within the OSTN pathophysiological range, the present findings from in vivo studies in conscious animals are consistent with comparable or higher affinity of OSTN for NPR‐C than ANP (Kita et al., 2009) and appreciably higher than we have observed between interactions of ANP and BNP in normal humans (Florkowski *et al.*, 1994; Hunt *et al.*, 1995). Notably, although raising plasma OSTN from 1 to 30 pmol/L in sheep increases plasma ANP, BNP and CNP, similar increments of either ANP or BNP in humans have no impact on plasma BNP or ANP, respectively (Hunt et al., 1995). Our study was not designed to examine the interactions of NPs and OSTN with NPR‐C at the cellular level in sheep. However, previous work in our laboratory (Smith *et al.*, 2000) shows that the binding affinity of ANP and ovine BNP to NPR‐C, using purified ovine lung tissue membranes, is high (*K*_d_ 8–16 pM) and similar to that reported in the rat (Suga *et al.*, 1992). Given the highly conserved aa sequence of rat and ovine OSTN, with the two binding domains being identical, it is likely that the molecular interactions reported by Kita et al. (2009) explain our findings. Clarifying the significance of these findings with respect to the potential role of OSTN in maintaining cardiovascular function (Miyazaki et al., 2018; Szaroszyk et al., 2022) and metabolism (Subbotina et al., 2015) will require knowledge of the tissues expressing OSTN, the local concentrations and potency of the relevant form of OSTN (5 or 10 kDa or higher) and their proximity to the appropriate receptors (NPR‐C, NPR‐A and NPR‐B). Although numerous studies show potent anti‐ inflammatory (Hu *et al.*, 2020; Miyazaki et al., 2018) and metabolic (Jin *et al.*, 2023; Zhang *et al.*, 2020) actions of OSTN, the tissues expressing OSTN and the tissue abundance of the protein in pathophysiological settings are unclear but important to clarify, in view of the very low concentrations of OSTN in the circulation. Cardiac expression of OSTN is controversial (Schafer *et al.*, 2018; Szaroszyk et al., 2022) but might be dependent on stress or overload, as evidenced by the impact of high‐intensity interval training (Jeremic *et al.*, 2020) or preconditioning (Harris *et al.*, 2023). Similar findings might underlie the regulation of OSTN in skeletal muscle (Kang *et al.*, 2024; Subbotina et al., 2015) and in adipocytes in obesity (Choi *et al.*, 2023; Nishizawa et al., 2004; Zhang et al., 2020). Collectively, these findings point to potentially important roles of OSTN in cardiovascular, muscle and adipose tissues, which need to be explored further using appropriate stressors in experimental animal models of pathological conditions, such as heart failure or obesity. Clearly, closer studies of the interactions of OSTN with NPs are now needed in humans where, in contrast to sheep, the affinity of BNP for NPR‐C is reduced.

The present observations linking OSTN (a naturally secreted NPR‐C ligand) with changes in NPs closely simulate our previous findings using the synthetic NPR‐C ligand, C‐ANP‐(4–23) (Charles, Espiner, Nicholls *et al.*, 1996), except that in the latter study stepped infusions, although inducing similar increases in ANP and BNP, had no significant haemodynamic effect even at the highest dose. Previous studies in normal sheep show that raising plasma concentrations of ANP or BNP to levels of ∼20–30 pmol/L result in a significant increase in cGMP and reduction in MAP and CO, without affecting sodium excretion (Charles, Espiner, Richards *et al.*, 1996). Overall, these results mimic the findings we observed when a similar NP increase was induced by OSTN, except that the consistent inhibition of aldosterone noted during exogenous NP infusions was not observed during OSTN, even at the highest dose. Given that the vasodilating effects were comparable in the two studies, and aldosterone concentrations prior to infusions of 2.5 pmol/kg/min were similar, the lack of impact of OSTN on aldosterone could relate to the location and abundance of NPR‐C. Notably, in human adrenal tissue NPR‐C expression is very low (Sarzani et al., 1996, 1999). The absence of any significant decline in renin during OSTN infusions (not measured in the report by Charles, Espiner, Richards *et al.*, 1996) might also be related to NPR‐C abundance, because no expression of NPR‐C mRNA (confirmed by real‐time PCR) was found in renin‐ and erythropoietin‐producing cells in mice (Heinl *et al.*, 2023). Although NP concentrations were similar in both studies, during the present experiments the OSTN‐induced increments in plasma NPs will be likely to reflect the local (and variable) displacement of NPs across a range of NPR‐C‐enriched vascular tissues. Also relevant here is the lack of significant increase in nephrogenous cGMP excretion during OSTN infusions, suggesting that renal NPR‐C abundance is lower than that expressed in the systemic vasculature and other organs. Previous studies have shown large increases (10‐fold) in nephrogenous cGMP in response to exogenous ANP infusions achieving plasma concentrations of ∼150 pmol/L in people (Hirata et al., 1990). Although NP concentrations during OSTN infusions are much lower, the lack of a significant cGMP response from renal tissues is consistent with reports of restricted expression of NPR‐C, in comparison to NPR‐A or NPR‐B, in healthy murine renal tissues (Heinl et al., 2023). Taken together, these findings provide further evidence supporting the importance of local tissue interactions of OSTN, NPs, NPR‐C and NPR‐A/B receptors in determining the functional outcome.

Absence of any natriuretic impact of activated OSTN–NP–NPR‐C signalling (also observed when NPs were raised to comparable levels by exogenous infusion; Charles, Espiner, Richards *et al.*, 1996) is unsurprising in light of the reduction in (renal) arterial pressure, which is likely to counterbalance any possible direct effect of NPs increasing sodium excretion in these normal sheep. In this context, it is interesting to note a recent report (Shao et al., 2021) showing that NPR‐C signalling, mediated by G_i_‐binding protein, regulates sodium reabsorption in distal convoluted tubules in male mice. Assuming an analogous situation in sheep, intense activation of NPR‐C by OSTN might be expected to amplify sodium reabsorption and to reduce adenyl cyclase and cAMP (Pagano & Anand‐Srivastava, 2001). Neither was observed in the present study. More specifically, nephrogenous cAMP was unchanged. However, the impact of NPR‐C activation on sodium reabsorption could be less in females and could be dependent on volume status (greater in settings of sodium depletion) (Shao et al., 2021). Clearly, further studies controlling for these variables are needed to assess better the role of OSTN in natriuresis in vivo.

To our knowledge, the metabolism of the 5 kDa OSTN has not been studied previously in vivo. Assuming that the immunoreactive form of OSTN remains intact during and after cessation of the 5 kDa peptide infusion, we found that OSTN MCR ranged from 2.55 ± 0.2 to 5.53 ± 0.7 L/min and was inversely proportional to OSTN dose. At comparable circulating concentrations (∼30 pmol/L), OSTN MCR (2.5 L/min) is approximately half that of the clearance rate of NPs (5.7, 7.5 and 4.7 L/min for ANP, BNP and CNP, respectively) (Charles, Espiner, Richards *et al.*, 1996). Consistent with this is the longer half‐life of OSTN (∼6 min) in comparison to NP (2–4 min), as reported previously in sheep (Charles, Espiner, Richards *et al.*, 1996). Notably, in the present study, plasma levels of all three NPs remained elevated for a sustained period after cessation of OSTN (see Figure 3), in keeping with a sustained reduction in NPR‐C capacity. Presumably, like bioactive NPs, OSTN is initially bound to NPR‐C, then internalized to interact with GEF‐H1 (a guanine nucleotide exchange factor) (Nishida et al., 2021). Furthermore, the barely detectable level of OSTN in the resting (basal) state, when plasma ANP concentration is ∼12–15 pmol/L, suggests that physiological levels of NPs have little or no impact on the OSTN concentration. Whether the peptide is a substrate for neprilysin degradation has not been studied formally, but modelling (on the basis of known aa sequences conferring hydrolysis) make it an unlikely target. Future studies of the activity and metabolism of larger forms, such as proOSTN (30–130), or the precursor (20 kDa) are needed, along with the role, if any, of smaller peptides cleaved from the mature peptide in vivo if either of the two motifs remains intact (Kita et al., 2009). Our HPLC analyses of immunoreactive forms need confirmation and do not exclude the presence of small amounts of the 10 kDa OSTN peptide in ovine plasma or small amounts of later‐eluting and smaller‐sized fragments that retain bioactivity. The HPLC analysis also reveals significant contributions to immunoreactivity by interfering matrix components, suggesting that authentic concentrations of OSTN in plasma in the resting (basal) state are even lower than reported here. Collectively, our findings emphasize the need for more precision in characterizing plasma forms of this pleotropic hormone that potentially have important roles in maintaining cardiovascular and metabolic health.

### 4.1 Study limitations

A limitation of the present study is that only the acute effects of OSTN treatment have been assessed. Although the study design, with its intensive integrated sampling and dual treatment arms, restricts study duration and prohibits study of tissue and activity at the cellular level, it permits a more complete initial assessment of effects and responses, which is especially important given that this is the first large‐animal investigation into the integrated cardiovascular, renal and neurohumoral responses to OSTN infusion.

The extensive hormone profiling (a notable strength of this study) did not include measurement of NTproANP or NTproBNP, assays of which were unavailable in sheep. Future study of these in an appropriate animal model will be important in validating the differential impact of OSTN on circulating concentrations of bioactive but not bioinactive NP forms.

## 5 CONCLUSION

We have reported a specific and sensitive RIA method for quantification of circulating OSTN. We have also quantified, for the first time, the dose‐dependent impact of OSTN on the plasma bioactive NP levels and associated changes in cardiovascular and renal function in normal sheep. The ability of OSTN, in tissues and in plasma, to impact NP concentrations in health or disease highlights the therapeutic potential of OSTN administration in cardiovascular disease and warrants further investigation.

## AUTHOR CONTRIBUTIONS

Nicola J. A. Scott, Christopher J. Charles, A. Mark Richards and Miriam T. Rademaker designed the study and obtained the funding. Nicola J. A. Scott and Timothy C. R. Prickett performed the experiments, analysed the data and prepared the figures. Nicola J. A. Scott and Eric A. Espiner wrote the manuscript. All authors reviewed the data and revised the manuscript. All authors approved the final manuscript and agree to be accountable for all aspects of the work in ensuring that questions related to the accuracy or integrity of any part of the work are appropriately investigated and resolved. All persons designated as authors qualify for authorship, and all those who qualify for authorship are listed.

## CONFLICT OF INTEREST

None.

## Data Availability

The underlying data and analysis in this manuscript are available upon reasonable request to the authors.
